# Combinatorial Drug Treatments Reveal Promising Anticytomegaloviral Profiles for Clinically Relevant Pharmaceutical Kinase Inhibitors (PKIs)

**DOI:** 10.3390/ijms22020575

**Published:** 2021-01-08

**Authors:** Markus Wild, Jintawee Kicuntod, Lisa Seyler, Christina Wangen, Luca D. Bertzbach, Andelé M. Conradie, Benedikt B. Kaufer, Sabrina Wagner, Detlef Michel, Jan Eickhoff, Svetlana B. Tsogoeva, Tobias Bäuerle, Friedrich Hahn, Manfred Marschall

**Affiliations:** 1 Institute for Clinical and Molecular Virology, Friedrich-Alexander University of Erlangen-Nürnberg (FAU), Schlossgarten 4, 91054 Erlangen, Germany; markus.wild@uk-erlangen.de (M.W.); jintawee.kicuntod@extern.uk-erlangen.de (J.K.); christina.wangen@uk-erlangen.de (C.W.); sabrina.wagner@uk-erlangen.de (S.W.); friedrich.hahn@uk-erlangen.de (F.H.); 2 Institute of Radiology, University Medical Center Erlangen, FAU, Palmsanlage 5, 91054 Erlangen, Germany; lisa.seyler@uk-erlangen.de (L.S.); tobias.baeuerle@uk-erlangen.de (T.B.); 3 Institute of Virology, Freie Universität Berlin, Robert-von-Ostertag-Straße 7-13, 14163 Berlin, Germany; luca.bertzbach@fu-berlin.de (L.D.B.); andele.conradie@fu-berlin.de (A.M.C.); benedikt.kaufer@fu-berlin.de (B.B.K.); 4 Institute for Virology, Ulm University Medical Center, Albert-Einstein-Allee 11, 89081 Ulm, Germany; detlef.michel@uniklinik-ulm.de; 5 Lead Discovery Center GmbH, Otto-Hahn-Str. 15, 44227 Dortmund, Germany; eickhoff@lead-discovery.de; 6 Institute of Organic Chemistry I, FAU, Nikolaus-Fiebiger-Straße 10, 91058 Erlangen, Germany; svetlana.tsogoeva@fau.de

**Keywords:** human cytomegalovirus, antiviral drugs, activity in vitro and in vivo, combinatorial drug analyses, pharmaceutical kinase inhibitors (PKIs), new synergistic combinations

## Abstract

Human cytomegalovirus (HCMV) is a human pathogenic herpesvirus associated with a variety of clinical symptoms. Current antiviral therapy is not always effective, so that improved drug classes and drug-targeting strategies are needed. Particularly host-directed antivirals, including pharmaceutical kinase inhibitors (PKIs), may help to overcome problems of drug resistance. Here, we focused on utilizing a selection of clinically relevant PKIs and determined their anticytomegaloviral efficacies. Particularly, PKIs directed to host or viral cyclin-dependent kinases, i.e., abemaciclib, LDC4297 and maribavir, exerted promising profiles against human and murine cytomegaloviruses. The anti-HCMV in vitro activity of the approved anti-cancer drug abemaciclib was confirmed in vivo using our luciferase-based murine cytomegalovirus (MCMV) animal model in immunocompetent mice. To assess drug combinations, we applied the Bliss independence checkerboard and Loewe additivity fixed-dose assays in parallel. Results revealed that (i) both affirmative approaches provided valuable information on anti-CMV drug efficacies and interactions, (ii) the analyzed combinations comprised additive, synergistic or antagonistic drug interactions consistent with the drugs’ antiviral mode-of-action, (iii) the selected PKIs, especially LDC4297, showed promising inhibitory profiles, not only against HCMV but also other α-, β- and γ-herpesviruses, and specifically, (iv) the combination treatment with LDC4297 and maribavir revealed a strong synergism against HCMV, which might open doors towards novel clinical options in the near future. Taken together, this study highlights the potential of therapeutic drug combinations of current developmental/preclinical PKIs.

## 1. Introduction

Human cytomegalovirus (HCMV), the prototype species of *Betaherpesvirinae*, represents an opportunistic human pathogen with a predominant, worldwide distribution. The seroprevalence in the adult human population lies in the range between 40% and 95% dependent on socio-geographic factors [1,2]. The infection with HCMV generally remains asymptomatic in immunocompetent individuals [3]. However, it can cause both morbidity and mortality in immunocompromised individuals, such as patients under antitumoral chemotherapy, stem cell/organ transplantation or coinfection with human immunodeficiency virus (HIV/AIDS). Importantly, congenital HCMV infection of the unborn is the main infection-based risk during pregnancy [4,5,6]. Congenital infection can cause a wide range of symptoms from mild to severe or even life-threatening in the unborn or infants, mainly manifesting as acute or late-onset embryonal developmental defects [7]. To date, a small number of anti-HCMV drugs have been approved for the prevention and control of infection, mostly comprising nucleoside/nucleotide or pyrophosphate analogs that intervene with the activity of viral genome replication, i.e., ganciclovir (GCV), its oral prodrug valganciclovir (VGCV), foscarnet (FOS) and cidofovir (CDV) [8,9]. By directly targeting the viral DNA polymerase and inhibiting the synthesis of viral DNA, specifically GCV and VGCV constitute the therapeutics of choice against HCMV infection. In 2017, letermovir (LMV, Prevymis®), which targets the viral terminase, was additionally approved and successfully used as an HCMV prophylaxis in recipients of hematopoietic stem cell transplantation [10,11]. LMV represents the first approved, mechanistically novel terminase inhibitor, which interferes with the packaging of viral DNA into mature capsids by the terminase complex. The specificity of LMV targeting translates to a very narrow spectrum of antiviral efficacy, being poorly effective against murine cytomegalovirus (MCMV) and ineffective against other human herpesviruses [12]. LMV is a promising drug candidate to be applied for further therapy options, possibly also including combination treatment. However, the currently approved anti-HCMV drugs face limitations, such as the induction of viral drug resistance and in many instances severe side effects. These side effects include nephrotoxicity, myelotoxicity and anemia, which limit therapeutic compatibility particularly in long-term treatments [13,14]. To tackle these issues of HCMV prevention and treatment, optimization of drug application schemes and novel targeting strategies are essential. For this reason, the development of mechanistically novel antiviral drug candidates and so far unexploited targeting strategies are the main goals of investigations (Figure S1). The kinase inhibitor maribavir (MBV) constitutes an anti-HCMV drug candidate which is in the advanced stages of development [15], currently being investigated in four phase III clinical trials (NCT02931539, NCT02927067, NCT00497796, NCT00411645). MBV targets the HCMV-encoded ortholog of cyclin-dependent kinases (CDKs), pUL97, which plays important roles in viral replication, particularly the HCMV nuclear capsid egress [16,17,18,19,20,21,22]. By preventing the phosphorylation of lamin A/C by pUL97, MBV blocks nuclear egress and thereby inhibits efficient viral replication and spread. MBV belongs to the class of pharmaceutical kinase inhibitors (PKIs) and, together with the aforementioned approved anti-HCMV drugs, to the class of direct-acting antivirals (DAAs), selectively targeting and inhibiting viral proteins. Hence, attempts should be made to broaden this repertoire by including host-directed antivirals (HDAs), such as artesunate-like drugs [23,24,25,26,27], as has been achieved in HIV-AIDS therapy with maraviroc and additional candidates [28,29]. Promising compounds for this host-directed approach are further PKIs targeting human CDKs. The selective CDK7 inhibitor LDC4297 exerts antiviral activity against HCMV and other herpesviruses in a nanomolar concentration range in vitro [30] and significantly reduces viral load in an MCMV mouse model [31]. Notably, no cellular antiproliferative or cytotoxic activity was detected in the relevant concentration range [30]; additionally, no adverse side effects were observed in mice [31]. HCMV inhibition by LDC4297 was already detectable at the immediate early phase of viral replication and may involve cell cycle modification through altered Rb phosphorylation [30]. Similarly, abemaciclib (ABE) represents another PKI with potential use as an HDA, but its anti-HCMV activity has not been assessed to date. ABE selectively inhibits cellular CDK4/6 and has been approved for treatment of advanced or metastatic breast cancer since 2017 [32]. In this study, we employed both in vitro and in vivo approaches to investigate the PKIs MBV, LDC4297 and ABE and further characterize their antiviral efficacies against HCMV, MCMV and additional herpesviruses.

In addition to new host-directed drugs, combination treatment represents another promising avenue to combat the emergence of drug resistance. A combination between GCV and FOS has been tested in patients with CMV infection and is recommended for CMV encephalitis [33,34,35,36], but in general, combination treatment against HCMV is not common. Combining antiviral drugs with different modes of action, however, may have significant advantages over monotherapy such as increased efficiency and decreased toxicity or side effects. In particular, HCMV combination treatments with both DAAs and HDAs might overcome the limitations of viral resistance and drug compatibility. In this study, drug combinations of two PKIs as well as combinations of one PKI and an approved direct-acting anti-HCMV drug were assessed. To this end, an affirmative parallel approach involving the Bliss independence checkerboard and Loewe additivity fixed-dose assays was applied to analyze the putative additional benefit of combinatorial treatment. Due to differences in the definition of synergism, in the employed calculations and the experimental procedures, these two approaches complement each other and can be used together for a comprehensive analysis of drug combinations. Based on the presented data, we report promising antiviral profiles for combinations of PKIs that may expand clinical therapy regimens to control HCMV infection.

## 2. Results and Discussion

### 2.1. Assessment of Antiviral Efficacies of a Selection of Clinically Relevant PKIs

Recently, we reported the strong antiviral properties of chemically distinct classes of HDAs. These were most intensely investigated for anti-HCMV effects but also against other viruses [23,24,25,37,38,39,40,41]. A broad spectrum of antiviral efficacy was revealed as a typical hallmark of HDAs, with PKIs repeatedly showing a promising profile of activity [30,31,42,43,44,45,46,47]. Building on this experience, we investigated several PKIs for antiviral activity here, focusing on a small number of drugs currently under preclinical (CDK7 inhibitor LDC4297) or clinical investigation (MBV) or approved for antitumoral therapy (ABE). In all cases, the assessment of anticytomegaloviral activities, i.e., determined in the present study for HCMV and MCMV, pointed to promising in vitro efficacies, with EC_50_ values in the low micromolar (ABE), submicromolar (MBV) or nanomolar range (LDC4297), respectively (Table 1). Moreover, broad-spectrum antiherpesviral activity (covering α-, β- and γ-herpesviruses) was assessed by including the animal oncogenic Marek’s disease virus (MDV) and the human oncogenic Epstein-Barr virus (EBV). Here, LDC4297 showed broad inhibitory activity against all analyzed viruses, in a consistently nanomolar concentration range. ABE likewise displayed a broad antiviral efficacy, consistently within a low micromolar concentration range. MBV, which represents an inhibitor of the HCMV kinase pUL97, exerted a pronounced anti-HCMV activity with a submicromolar EC_50_ value of 0.56 ± 0.60 µM, but no in vitro activity against other herpesviruses. Only a concentration of 30 µM of MBV resulted in some weak antiviral effect on EBV in vitro, which was only partly in line with the ongoing clinical investigations of the EBV-directed MBV efficacy.

### 2.2. First Characterization of Abemaciclib as a New Investigational Antiviral Drug

**Figure 1:**
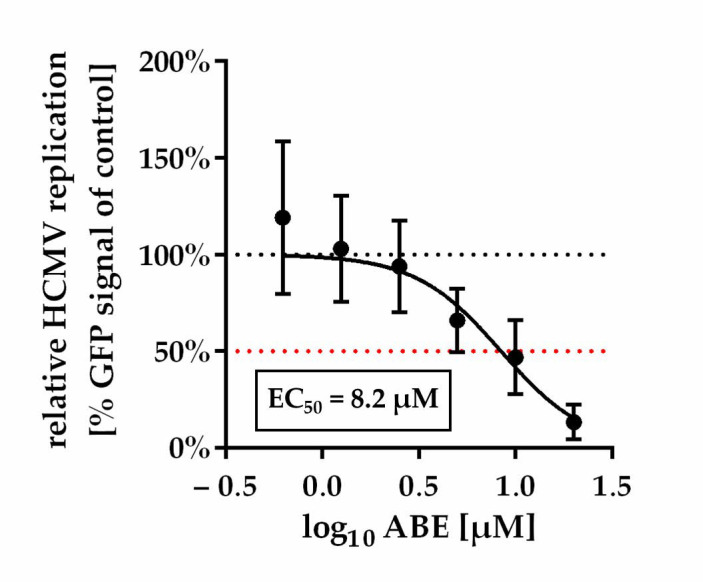
Dose-response curve of ABE against HCMV in vitro. Data are presented as mean relative HCMV replication compared to control ±SD over three independent experiments each consisting of biological duplicates.

**Figure 2:**
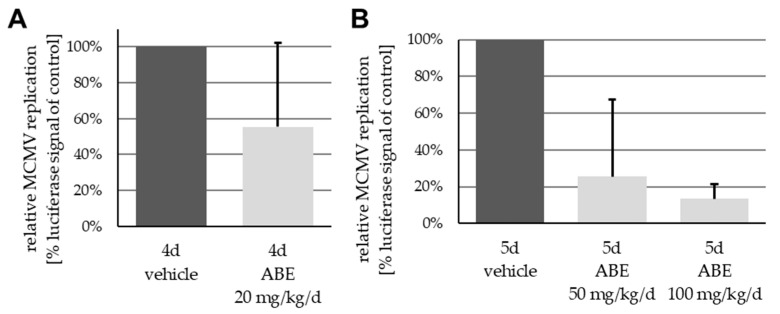
In vivo antiviral drug dosage assessment: ABE-mediated concentration-dependent reduction of spleen-specific MCMV viral load. Oral application of ABE in dosages of (**A**) 20 mg/kg/d over 4 days and (**B**) 50 mg/kg/d or 100 mg/kg/d over 5 days, resulting in a reduction in viral load of 44%, 74% or 87%, respectively, compared to vehicle-treated animals. Data are presented as mean + SD of viral replication across five animals per group as measured using in vitro luciferase assay performed on spleen homogenates.

While a substantial characterization of the antiviral properties of MBV and LDC4297 in vitro and in vivo has been reported [30,31,42,47,48,49,50,51,52,53,54], any similar antiviral activity of ABE has not yet been assessed. To this end, the in vitro and in vivo inhibitory properties of ABE were determined against HCMV and MCMV, respectively. For both viruses, a concentration-dependent decline of viral replication was demonstrated, in the case of HCMV in vitro (Figure 1) and MCMV in vivo (Figure 2). Note the slight, so far mechanistically unexplained, proviral effect of low concentrations of ABE (<1 µM) in vitro (Figure 1), which was reproducibly observed in independent experimental replicates using varied multiplicities of infection (MOIs). This effect was not reflected by the in vivo experimentation, in which all analyzed dosages, i.e., 20, 50 and 100 mg/kg/d, resulted in a detectable inhibition of MCMV replication in the spleen (Figure 2), although variability between animals was relatively high.

**Figure 3:**
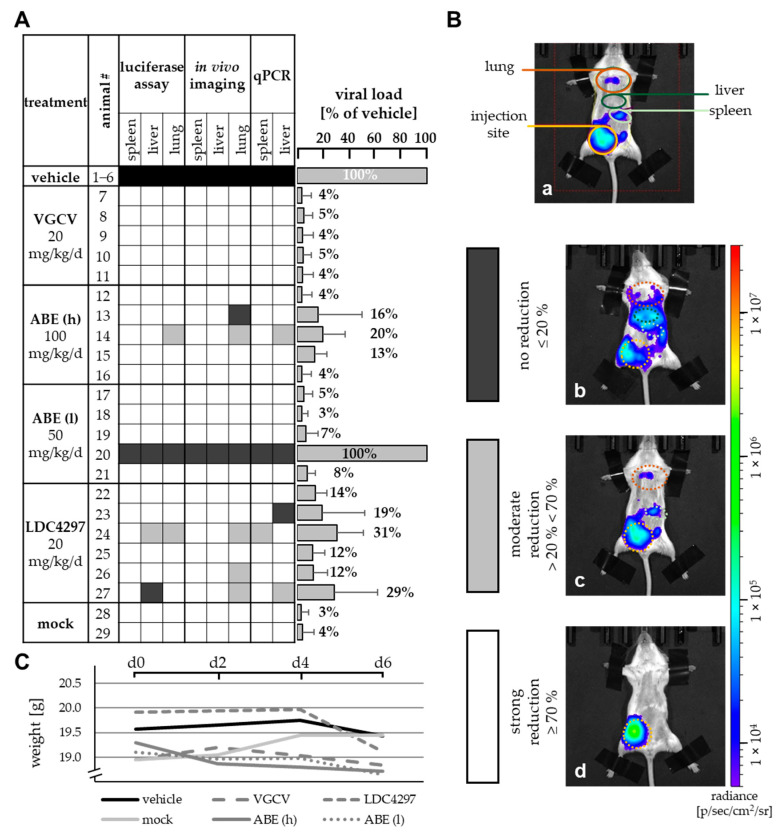
Reduction of viral load by PKI treatment. (**A**) Viral load in spleen, liver and lung was measured using in vitro luciferase assay, in vivo luciferase imaging and viral genome-specific qPCR and was compared to the mean value of viral load of six vehicle-treated mice. No, moderate or strong reduction is indicated by black, grey or white colored boxes, respectively, as indicated in panel B. Grey bars on the right indicate mean values + SD of viral load for each animal in all eight analyzed organs/methods (see also percentages given above of each bar). (**B**) Exemplary pictures of in vivo luciferase imaging. Mice were fixed, sedated and injected with luciferin solution. Organ-specific luciferase activity was measured according to regions of interest marked in image a. Pictures show exemplary animals for the categories used in panel A, i.e., no reduction (image b), moderate reduction (image c), strong reduction (image d). (**C**) Weight of treated animals as determined on days 0, 2, 4 and 6. Data are given as mean weight in each treatment group. VGCV, valganciclovir.

This anti-MCMV activity of ABE in vivo was further evaluated via a comparison with reference drug valganciclovir (VGCV) and LDC4297, which had been analyzed in an immunodeficient mouse model previously [31]. In the present approach, MCMV intraperitoneal infection was performed with immunocompetent Balb/c mice and drugs were administered via oral gavage (Figure 3). Note that in this regimen, VGCV can be considered the gold standard of oral treatment, showing the most efficient reduction of viral load (Figure 3A, right panel). ABE at a higher dose of 100 mg/kg/d (ABE h) and LDC4297 (20 mg/kg/d) showed substantial levels of inhibition, while the lower ABE dose of 50 mg/kg/d (ABE l) showed limited efficacy, with one animal even displaying lack of reduction of viral load (Figure 3A, #20). Quantities of viral load are expressed as a mean of all eight analyzed organs/methods for each animal, given in percentage of the mean vehicle control (Figure 3A, #1–6, set as 100%). The individual parameters determined in this setting were based on in vitro luciferase assays performed on homogenates of three different organs, (spleen, liver and lung), qPCR assays using DNA extractions of two organs, (spleen and liver), as well as luciferase measurement via in vivo imaging (spleen, liver and lung; Figure 3B). As a control measurement of drug compatibility, none of the animals showed a reduction of body weight below 18.5 g (92.5% of maximum) under these treatment conditions (Figure 3C). Combined, the data illustrate the anti-MCMV activity of ABE in this animal model with the highest efficacy observed for 100 mg/kg/d. 

### 2.3. Assessment of Antiviral Drug Combinations Using an Approach with Both Bliss Independence Checkerboard and Loewe Additivity Fixed-Dose Assays

**Figure 4:**
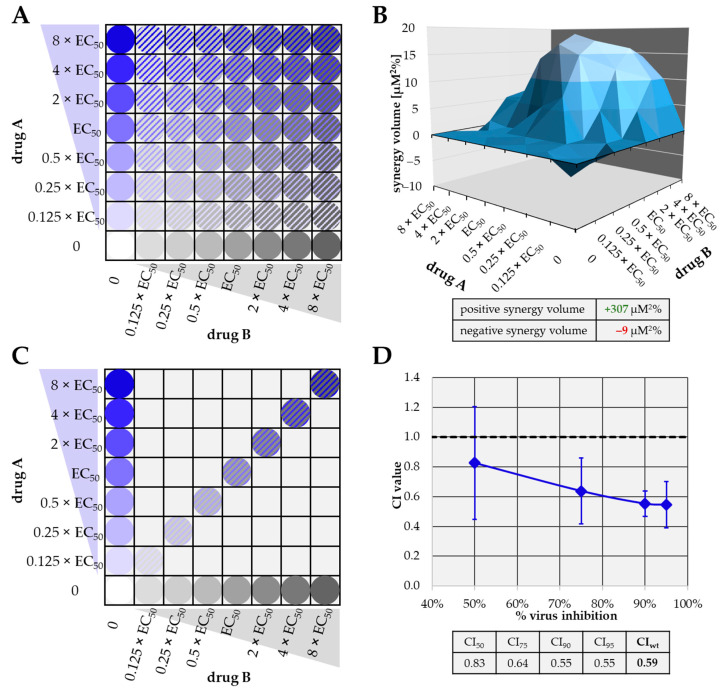
Characteristics of the Bliss independence checkerboard (**A**,**B**) and the Loewe additivity fixed-dose assays (**C**,**D**) adapted to the assessment of anticytomegaloviral drug combinations in vitro. (**A**) Schematic depiction of exemplary concentration combinations of drugs A (blue) and B (grey) employed in the Bliss independence checkerboard assay. (**B**) Exemplary synergistic result of a checkerboard assay. Positive synergy volume (volume above the 0 plane, green) as well as negative synergy volume (volume below 0 plane, red) are given in the table below. (**C**) Schematic depiction of exemplary concentration combinations of drugs A (blue) and B (grey) employed in the Loewe additivity fixed-dose assay. (**D**) Exemplary synergistic result of a fixed-dose assay. CI values at 50%, 75%, 90% and 95% virus inhibition as well as the calculated weighted CI value (CI_wt_) are given in the table below. CI_wt_ was calculated as (1 × CI_50_ + 2 × CI_75_ + 3 × CI_90_ + 4 × CI_95_)/10. Note that the data shown in panels B and C are exemplary data from Figure 5 and Figure 6. CI, combination index.

The efficacy of combinatorial antiviral drug treatments in vitro was assessed using the two most approved methods in an affirmative parallel approach, in order to achieve highly reliable quantitative results (Figure 4). Volumes of drug synergy, additivity or antagonism (measured as synergy volume in µM^2^%) were determined according to the Bliss independence checkerboard assay [55], and combination indices (CIs) were calculated according to the Loewe additivity fixed-dose assay [56].

For the Bliss independence model, serial drug dilutions were analyzed in checkerboard-like comprehensive combinations. This approach is labor-intensive, but has the advantage that minor inaccuracies of preceding EC_50_ value determinations can be compensated by the relatively large amount of data (Figure 4A). Raw data were analyzed using MacSynergy II software (The University of Alabama at Birmingham, Birmingham, AL, USA) developed by Prichard and Shipman (1990, [55]). The program uses the independent-effects definition of additive interactions, meaning that theoretical additive interactions are calculated from the dose-response curves for each drug alone. This calculated additive surface is then subtracted from the experimentally determined dose-response surface to reveal regions of non-additive activity. The resulting surface appears as a horizontal plane at 0 µM%^2^ synergy volume (Figure 4B) when a drug combination is merely additive (no antagonistic or synergistic interaction). Any peaks above or below this plane of additivity indicate synergism or antagonism, respectively. The volumes of the peaks/depressions were expressed as the respective synergy/antagonism volumes [µM^2^%], and types of drug interaction were defined as follows: values below −100, strongly antagonistic; −100 to +50, additive; +50 to +100, moderately synergistic; above +100 strongly synergistic [57,58] (Table 2).

For Loewe additivity evaluations, a lower number of drug concentration pairs was analyzed. This approach is dependent on the preceding determination of EC_50_ values in a very accurate way and has the advantage of largely reducing the number of test samples (Figure 4C). Raw data were calculated using CompuSyn software (Version 1.0 [59], ComboSyn, Inc., Paramus, NJ, USA) based on the procedure developed by Chou and Talalay (1984, [56]). Here, the program first converts the dose-effect curves for each drug or drug combination to median effect plots, before CI values for the combinations are extrapolated at 50% (CI_50_), 75% (CI_75_), 90% (CI_90_) and 95% (CI_95_) virus inhibition from actual calculated CIs (Figure 4D). Here, a CI value of 1 implies additive interaction, <1 synergistic and >1 antagonistic. A weighted CI (CI_wt_) was calculated from the four aforementioned CI values, giving higher weight with increasing virus inhibition. Synergy, antagonism or additivity indicated by the CI_wt_ were defined as follows: values <0.1 to 0.3, strongly synergistic; 0.3 to 0.7, synergistic; 0.7 to 0.85, moderately synergistic; 0.85 to 0.9, slightly synergistic; 0.90 to 1.10, (nearly) additive; 1.10 to 1.20, slightly antagonistic; 1.20 to 1.45, moderately antagonistic; 1.45 to 3.3, antagonistic; 3.3 to >10, strongly antagonistic [60] (Table 3). The two independent methods were applied for the drug combinations analyzed in this study as an affirmative parallel approach [55,56,59,60], and comprehensive data sets are given below.

### 2.4. Identification of Additive, Synergistic or Antagonistic Types of Drug Interaction for Various Combinations of PKIs and Other Antiviral Compounds

**Figure 5:**
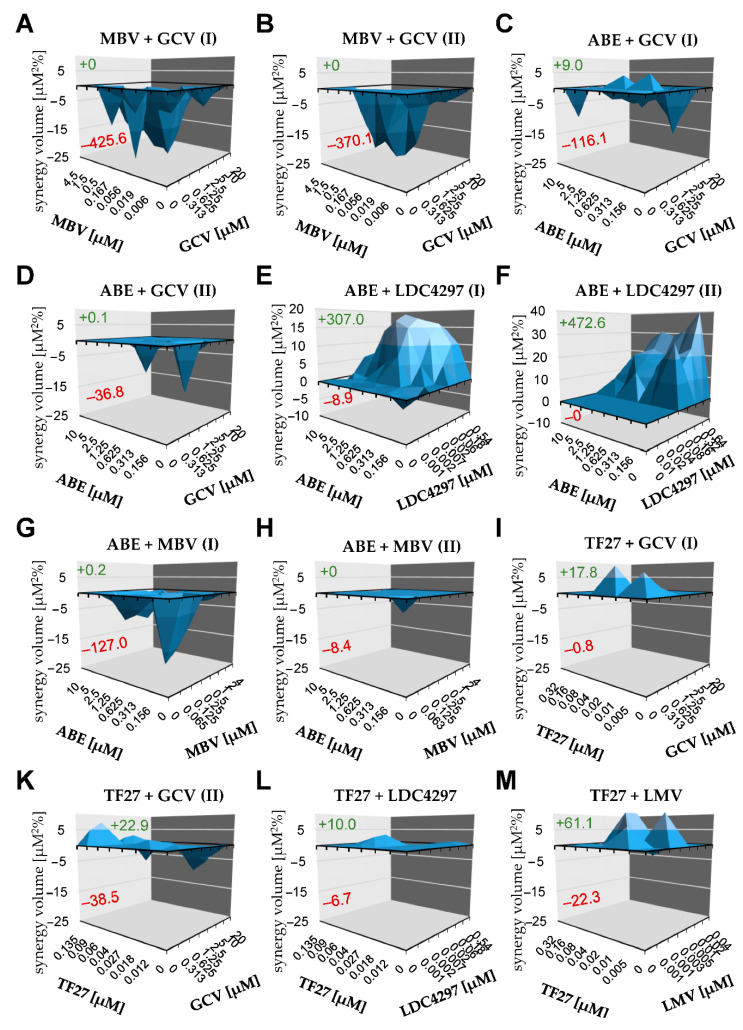

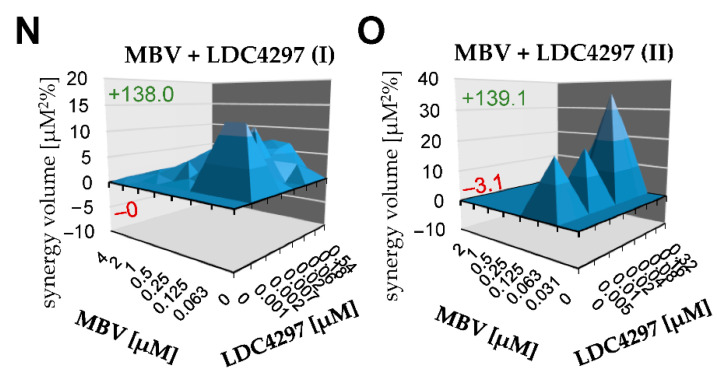
Bliss independence checkerboard assay results (HCMV-infected HFFs in all cases). (**A**) MBV + GCV (replicate I); (**B**) MBV + GCV (replicate II); (**C**) ABE + GCV (replicate I); (**D**) ABE + GCV (replicate II); (**E**) ABE + LDC4297 (replicate I); (**F**) ABE +LDC4297 (replicate II); (**G**) ABE + MBV (replicate I); (**H**) ABE + MBV (replicate II); (**I**) TF27 + GCV (replicate I); (**K**) TF27 + GCV (replicate II); (**L**) TF27 + LDC4297; (**M**) TF27 + LMV; (**N**) MBV + LDC4297 (replicate I); (**O**) MBV + LDC4297 (replicate II).

The series of chosen drug combinations were assessed using the Bliss independence checkerboard assay, using primary human foreskin fibroblasts (HFF), in the HFF/HCMV in vitro infection model (Figure 5). The test settings comprised combinations between two PKIs themselves, i.e., maribavir (MBV), abemaciclib (ABE) and LDC4297 (panels C, F, G and H), or between one PKI and a mechanistically distinct antiviral reference drug such as ganciclovir (GCV), letermovir (LMV) or trimeric artesunate (TF27; [61,62,63]) (panels A, B, D, E and I). Additional combinations between the latter reference drugs were also analyzed in parallel (panels K, L and M). Most combinations showed additive effects, but the combination MBV + GCV showed antagonism, as expected (MBV inhibits viral kinase pUL97, while GCV needs activating phosphorylation through pUL97). Using this Bliss independence checkerboard assay, most importantly, two examples of strong synergism were newly identified with the combinations ABE + LDC4297 and MBV + LDC4297 (Table 2). This result argues for a very promising potential of these PKIs in anti-HCMV combinatorial drug development.

**Figure 6:**
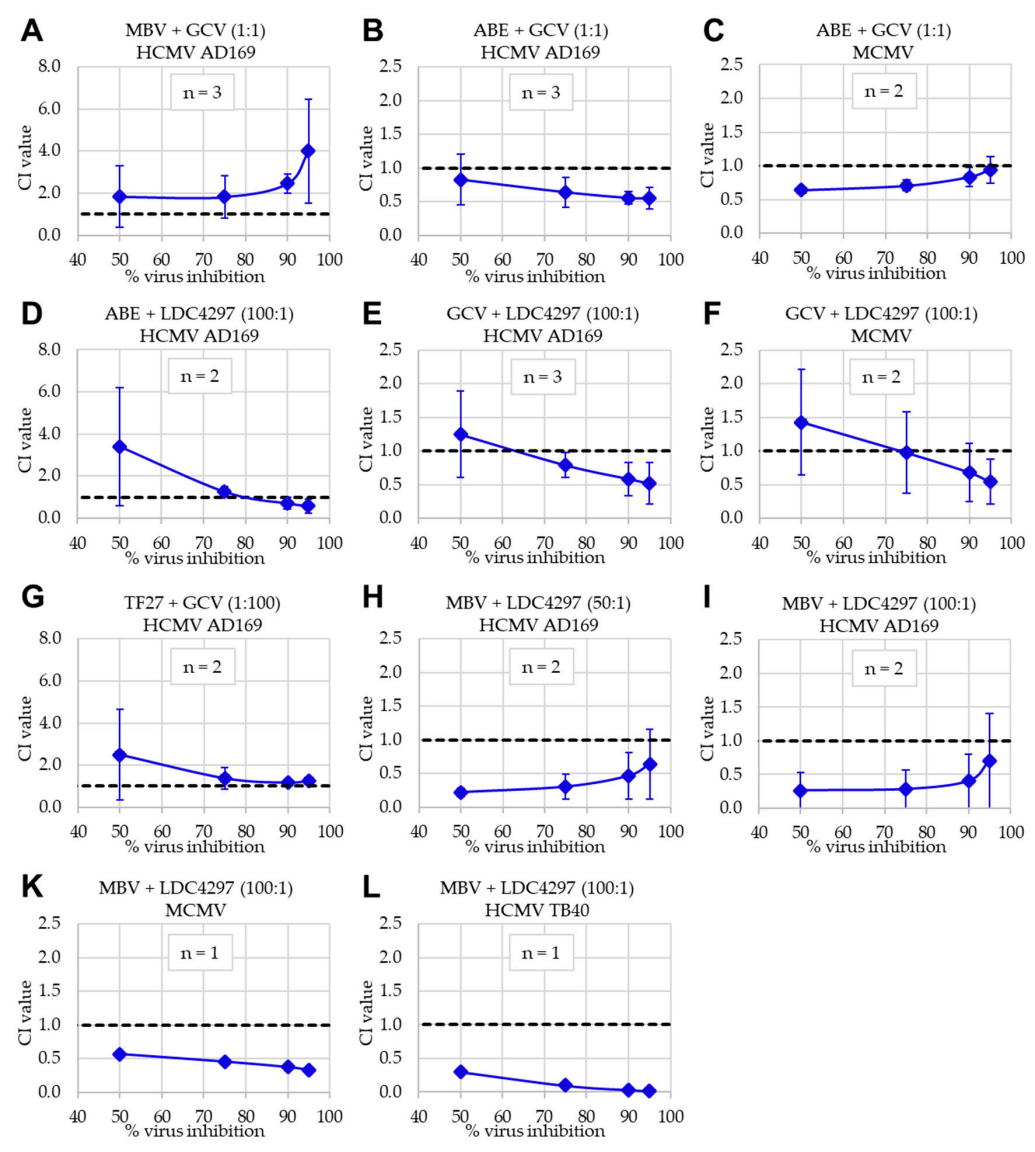
Loewe additivity fixed-dose assay results. Numbers in brackets indicate fixed ratio of drugs in the respective combination. (**A**) MBV + GCV (1:1) against HCMV in HFFs; (**B**) ABE + GCV (1:1) against HCMV in HFFs; (**C**) ABE + GCV (1:1) against MCMV in MEFs; (**D**) ABE + LDC4297 (100:1) against HCMV in HFFs; (**E**) GCV + LDC4297 (100:1) against HCMV in HFFs; (**F**) GCV + LDC4297 (100:1) against MCMV in MEFs; (**G**) TF27 + GCV (1:100) against HCMV in HFFs; (**H**) MBV + LDC4297 (50:1) against HCMV in HFFs; (**I**) MBV + LDC4297 (100:1) against HCMV in HFFs; (**K**) MBV + LDC4297 (100:1) against MCMV-UL97 in MEFs. (**L**) MBV + LDC4297 (100:1) against HCMV TB40 in ARPE-19 cells. Data are presented as mean CI values ± SD, extrapolated to 50%, 75%, 90% and 95% virus inhibition across the number of individual experiments (*n* = 1 to *n* = 3).

Applying the Loewe additivity fixed-dose assay in the parallel approach, these drug combinations were assessed in both human and murine in vitro infection models, i.e., HFF/HCMV and primary mouse embryonic fibroblasts (MEF)/MCMV (Figure 6). In the latter setting, the combination of MBV + LDC4297 was adapted to the murine system by using a recombinant MCMV in which the coding region for kinase pM97 was replaced by that for pUL97, thereby rendering the virus MBV-sensitive [64]. Here, the profile of effects obtained for PKI combinations and additional drug combinations was highly consistent with that obtained with the Bliss independence checkerboard assay. Also in this approach (Loewe), antagonism was confirmed for MBV + GCV (HCMV) and synergy was confirmed for MBV + LDC4297 (Figure 6, panels A, H, I, K and L; Table 2; HCMV AD169, HCMV TB40 and MCMV-UL97). To confirm the synergistic interaction in a second HCMV in vitro infection model, the combination of MBV + LDC4297 was assessed in epithelial ARPE-19 cells infected with an EYFP-expressing HCMV TB40 reporter virus. Importantly, the strong synergistic interaction of this drug combination was also detected in this setting, thus showing that this antiviral activity was not restricted to the laboratory strain AD169 in HFFs. In addition, moderately synergistic to clearly synergistic effects were measured for further drug combinations, i.e., ABE + GCV (HCMV and MCMV) and GCV + LDC4297 (HCMV and MCMV). Other combinations, including ABE + LDC4297, were found additive in this approach (Table 3). Thus, the results obtained through combinatorial drug assessment using two affirmative methods showed a high degree of consistency and synergistic effects could be identified for specific drug combinations including the PKIs.

### 2.5. Conclusions: Identification of Three New Synergistic Drug Combinations, Particularly the Most Efficient Combination between the Two PKIs MBV and LDC4297

**Figure 7:**
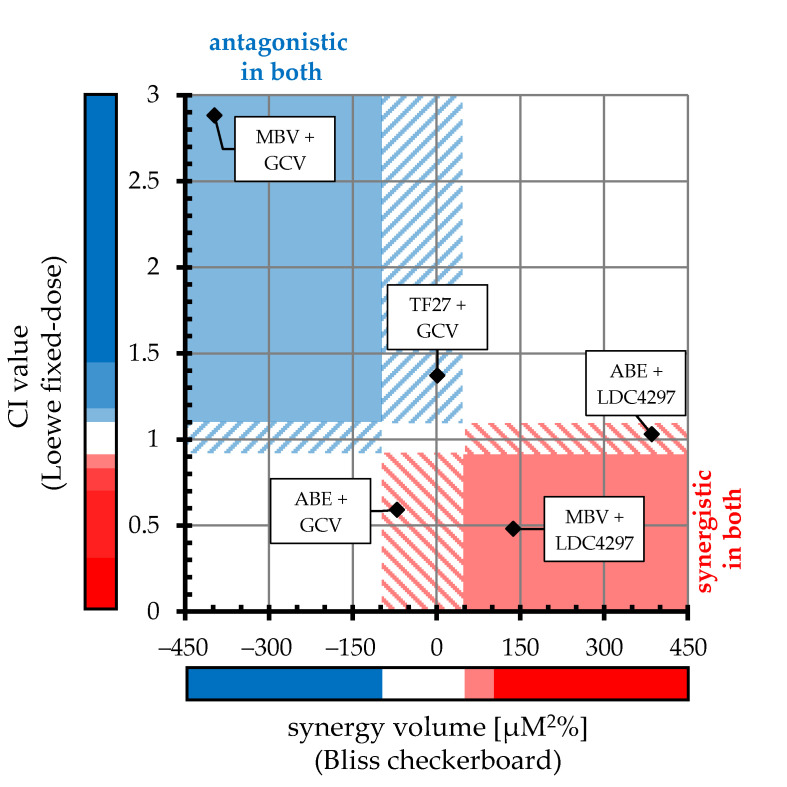
Comparative alignment of HCMV-specific Loewe fixed-dose and Bliss checkerboard results. X-axis value of each drug combination indicates mean synergy volume across checkerboard assays (positive and negative synergy volumes were added together to acquire one plottable value). Y-axis value indicates mean CI_wt_ across fixed-dose assays. Colored bars represent ranges of antagonistic (blue), additive (white) and synergistic (red) interactions for each approach (specified in Table 2 and Table 3). Solid blue/red fields within the chart indicate overlapping antagonistic/synergistic range of both approaches, respectively; dashed blue/red areas designate antagonistic or synergistic ranges in one method with additive values in the other method.

By applying the Loewe fixed-dose and Bliss checkerboard assays in this study, a consistent profile of antiviral effects of combinatorial treatment was obtained. In Figure 7, CI values and synergy volumes of the two methods are plotted against each other, and the type of drug combination is designated by color: antagonistic (blue), additive (white) and synergistic (red). In this illustration, solid blue/red fields indicate the overlapping antagonistic/synergistic ranges of both approaches, while dashed blue/red areas designate antagonistic or synergistic range in one method with additive values in the other method.

Combined, the results led to the following conclusions: (i) antagonistic interaction was found for the MBV + GCV combination (both methods); (ii) synergistic interaction was found for the MBV + LDC4297 combination (both methods); and (iii) intermediate effects of interaction were found for TF27 + GCV (additive), ABE + GCV and ABE + LDC4297 (additive-to-synergistic). These results are primarily based on the analysis of HCMV AD169 and were partly confirmed for HCMV TB40 and MCMV as described in detail above. Our findings specifically highlight a very promising potential of the synergistic interaction between the two PKIs MBV and LDC4297 (Figure 7). MBV is presently under clinical investigation in four phase III studies specific for HCMV infection and LDC4297 successfully passed preclinical investigations (unpublished data). The postulated mode of action of LDC4297, i.e., a selective inhibition of the various CDK7 activities relevant for its anti-HCMV efficacy, was experimentally addressed via an initial Western blot (Wb) analysis of a typical CDK7 substrate, the retinoblastoma protein (Rb). After a 9-h treatment of HFFs with 1 µM of LDC4297, cellular lysates were prepared and subjected to a standard Wb analysis using phospho-dependent and phospho-independent Rb antibodies, indicating a slight reduction of T821-phosphorylated Rb, while the phospho-independent Rb level remained unaltered (data not shown). In addition, an LDC4297-mediated inhibition of Rb phosphorylation at S807/811 in HCMV-infected HFFs has been reported previously [30].

It should be emphasized that the present data, based on both in vitro and in vivo infections, as well as combinatorial treatment approaches, consistently show antiviral activity of PKIs for those herpesviruses analyzed. Additionally, the observed effects of drug interaction strongly support the postulate that two drugs with different antiviral modes of action and compatible targeting mechanisms possess a substantial potential to act in an additive or even true synergistic manner. In this investigation, drug synergy was defined on the basis of two methods, both well-established for the assessment of combinatorial treatment. The presented data clearly illustrated significant levels of antagonistic interaction as measurable using either of the two methods for two drugs targeting molecular mechanisms dependent on each other, e.g., MBV and GCV (MBV acts as an inhibitor of viral kinase pUL97, while the inhibitory activity of GCV is dependent on active pUL97). Both drugs possessing complex inhibitory mechanisms (e.g., artesunate-like compounds such as TF27 or others), but exhibiting compatible targeting profiles, demonstrated additive or even synergistic effects of interaction (TF27 + GCV, TF27 + LDC4297 or TF27 + LMV). Similarly, for the combinations between monoselective PKIs, i.e., directed to CDK4/6, CDK7 or the viral CDK ortholog pUL97, examples of additive effects were obtained (such as ABE + MBV or ABE + LDC4297). This likewise holds true for many other mechanistically distinct, additive drug combinations, including GCV, which may nevertheless prove beneficial as new HCMV treatments and therefore merit a closer investigation in clinical approaches. As the computer-assisted prediction of a truly synergistic drug combination, however, is almost impossible, an experimental assessment based on the two applied approaches is crucial for ensuring reliability [26,27,65]. In the present study, we identified strong synergism between the two PKIs MBV and LDC4297, constituting a very promising experimental outcome of combinatorial assessment. Thus, these novel findings strongly suggest further clinical developmental analysis of PKI drug combinations as future options for anti-HCMV prophylaxis and treatment.

## 3. Materials and Methods

### 3.1. Cells and Viruses

Primary human foreskin fibroblasts (HFFs, derived from clinical samples, Children’s Hospital, Erlangen, Germany) were grown in Eagle’s Minimal Essential medium (MEM) supplemented with 1 × GlutaMAX^TM^ (both Thermo Fisher Scientific, Waltham, MA, USA), 10 μg/mL gentamicin and 10% fetal bovine serum (FBS, Capricorn, Ebsdorfergrund, Germany). Mouse embryonic fibroblasts (MEFs, ATCC, Manassas, VA, USA) were cultivated in Dulbecco’s modified Eagle’s medium (DMEM), supplemented with 1 × GlutaMAX^TM^ (both Thermo Fisher Scientific, Waltham, MA, USA), 10 μg/mL gentamicin and 10% fetal bovine serum (FBS, Capricorn, Ebsdorfergrund, Germany). ARPE-19 cells (repository of William D. Rawlinson, The Prince of Wales Hospital, Sydney, Australia) were cultivated in DMEM, supplemented with 1 × GlutaMAX^TM^ (both Thermo Fisher Scientific, Waltham, MA, USA), 10 μg/mL gentamicin and 10% fetal bovine serum (FBS, Capricorn, Ebsdorfergrund, Germany). Akata-BX-1 EBV-GFP producer cells [66] were grown in RPMI 1640 medium, supplemented with 1 × GlutaMAX^TM^ (both Thermo Fisher Scientific, Waltham, MA, USA), 10 μg/mL gentamicin and 10% fetal bovine serum (FBS, Capricorn, Ebsdorfergrund, Germany). Cultured cells were maintained at 37 °C, 5% CO_2_ and 80% humidity. All cell cultures were regularly monitored for absence of mycoplasma contamination (Lonza™ Mycoalert™, Thermo Fisher Scientific, Waltham, MA, USA). Recombinant HCMV AD169 expressing green fluorescent protein (AD169-GFP, [67]), recombinant HCMV TB40 expressing enhanced yellow fluorescent protein (TB40-EYFP, [68]) and recombinant MCMV Smith-GFP were used for in vitro replication assays. A recombinant MCMV, in which the coding region for MCMV kinase pM97 was replaced by that for HCMV pUL97 [64], was used in a plaque reduction assay (PRA). In vivo experiments were performed using the luciferase-tagged MCMV Smith strain (MCMV-del157luc) [69].

### 3.2. Antiviral Compounds

Antiviral drugs were obtained from the following sources: abemaciclib (ABE) and maribavir (MBV; MedChemExpress, Monmouth Junction, NJ, USA); TF27 (Vichem Chemie Research Ltd., Budapest, Hungary) [30,61,62]; letermovir (LMV; Cayman Chemical Company, Ann Arbor, MI, USA); valganciclovir (VGCV; Ratiopharm, Ulm, Germany); LDC4297 (Lead Discovery Center GmbH, Dortmund, Germany). Stock aliquots were prepared in DMSO and stored at −20 °C.

### 3.3. Assessment of Antiviral Efficacy Against EBV

Akata-BX-1 EBV-GFP producer cells [66] were grown in RPMI 1640 medium and split 1:2 (vol/vol) one d before experimentation, then seeded in 24-well plates at 1 × 10^6^ cells/well and incubated with serial dilutions of antiviral compounds. After 3 d, EBV-GFP-positive cells were monitored using fluorescence microscopy for cell viability, GFP expression and virus-induced signs of a cytopathic effect (CPE). Cell lysis was performed under conditions described below for the HCMV AD169-GFP-based replication assay (3.6., 3.7) and lysates were subjected to automated quantitative GFP fluorometry in quadruplicate measurements as reported previously [67].

### 3.4. Assessment of Antiviral Efficacy Against MDV

Chicken embryonic cells were prepared from 11-day old specific-pathogen-free (SPF) chicken embryos (VALO BioMedia, Osterholz-Scharmbeck, Germany) as described previously [70] and cultivated in MEM (PAN Biotech, Aidenbach, Germany) supplemented with 10% fetal bovine serum and antibiotics (100 U/mL penicillin and 100 µg/mL streptomycin). A highly virulent MDV GFP-expressing reporter virus was used as described previously [71]. Virus replication and EC_50_ values were assessed using PRA and qPCR in the presence or absence of antiviral compounds. 10^6^ cells were infected with 100 plaque-forming units (PFU) and analyzed at 5 d post-infection (p.i.) MDV-induced plaque formation was counted under the microscope in triplicates, and MDV genome copies were quantitated using qPCR with primers and a probe specific for the viral gene ICP4. Viral copy numbers were normalized against chicken iNOS genome copies to obtain the viral load per cell [72]. All experiments were performed as three independent replicates.

### 3.5. Bliss Checkerboard Assay Adapted to HCMV-GFP In Vitro Infection

Bliss additivity was assessed using an adapted protocol of the HCMV GFP-based replication assay described previously [30,67]. HFFs were seeded at 1.2 × 10^4^ cells/well in 96-well culture plates (3 plates per assay) and infected the next day with HCMV AD169-GFP [67] at an MOI of 0.25 (i.e., 25% GFP-forming dose of a multi-round infection measured at 7 d p.i.) or remained mock-infected. After virus adsorption of 90 min, the inoculum was replaced by medium supplemented with a matrix of drug combinations in different concentration ratios, solvent control or medium for mock-infected wells. Standard protocol tested a matrix of 8 × 8 (see Figure 4A). All infections were performed in biological triplicates. Cells were lysed by the addition of 100 µL lysis buffer/well 7 d p.i.; cell suspensions were mixed and transferred to another 96-well plate. Centrifugation was performed at 3000 rpm for 15 min and clear lysates were subjected to automated GFP quantitation in a Victor X4 microplate reader (PerkinElmer, Waltham, MA, USA). Measured values were entered into MacSynergy II software ([55] The University of Alabama at Birmingham, Birmingham, AL, USA) and results presented as surface graphs illustrating synergy volume with a 95% confidence interval over the three biological replicates.

### 3.6. Loewe Fixed-Dose Assay Adapted to HCMV-GFP/HCMV-EYFP In Vitro Infection

Loewe additivity was assessed using an adapted protocol of the HCMV GFP-based replication assay described previously [30,67]. HFFs were seeded at 1.6 × 10^5^ cells/well in 12-well culture plates (4 plates per assay) and infected the next day with HCMV AD169-GFP [67] or HCMV TB40-EYFP [68] at an MOI of 0.25 (i.e., 25% GFP/EYFP-forming dose of a multi-round infection measured at 7 d p.i.). After virus adsorption of 90 min, the inoculum was replaced by medium supplemented with single compound, compound combination or solvent control. Standard protocol tested a serial dilution of 6 concentrations/single compound starting with app. 4–8 × EC_50_ and a serial dilution of 8 concentrations of the combination, starting with the highest concentrations of both single dilution series. All infections were performed in biological duplicates. Cells were lysed by the addition of 200 µL lysis buffer/well 7 d p.i.; cell suspensions were mixed and transferred to a 96-well plate. Centrifugation was performed at 3000 rpm for 15 min and clear lysates were subjected to automated GFP/EYFP quantitation in a Victor X4 microplate reader (PerkinElmer, Waltham, MA, USA). Antiviral efficacy (mean of duplicate measurement of biological duplicates) was expressed as the percentage of solvent control and entered into CompuSyn software (Version 1.0 [59]; ComboSyn, Inc., Paramus, NJ, USA). Only experiments with an r value > 0.90 and EC_50_ values close to previously determined concentrations were accepted. CI values extrapolated at 50, 75, 90 and 95% virus inhibition are plotted in Figure 6, if *n* > 1 as mean ± SD across experiments.

### 3.7. Loewe Fixed-Dose Assay Adapted to MCMV-GFP/MCMV-UL97 In Vitro Infection

Drug interactions in anti-MCMV efficacy were assessed in MEFs in a GFP-based approach similar to the HFF/HCMV setting using MCMV-GFP. Deviations were the number of seeded cells (1.5 × 10^5^ cells/well), as well as the time period in between infection and harvest (5 days). For assessing drug efficacy against the recombinant MCMV-UL97, an in vitro infection PRA was performed in MEFs. Four 12-well culture plates were seeded with MEFs at 1.5 × 10^5^ cells/well and infected one day later with MCMV-UL97. After virus adsorption of 90 min, the inoculum was replaced by medium supplemented with single compound, compound combination or solvent control, as well as 0.3% agarose (Serva, Heidelberg, Germany). A serial dilution of 6 concentrations/single compound starting with app. 4 × EC_50_ and a serial dilution of 8 concentrations of the combination, starting with the highest concentrations of both single dilution series, were used. All infections were performed in biological duplicates. Agarose was removed 5 d p.i., the cell layer was fixed and stained with 1% crystal violet (Serva, Heidelberg, Germany) in 20% ethanol solution, and plaques were counted under the microscope. Antiviral efficacy (mean of biological duplicates) was expressed as percentage of solvent control and entered into CompuSyn software (Version 1.0 [59]; ComboSyn, Inc., Paramus, NJ, USA).

### 3.8. Animal Experimentation

Female Balb/cAnNCrl mice (6 weeks of age) were purchased from Charles River Laboratories (Wilmington, MA, USA), maintained under specific pathogen-free conditions and utilized between 7 and 11 weeks of age. Caging was performed in groups of 3 mice and body weight was monitored on days 0, 2, 4 and 6 post-infection (p.i.). Animals were infected with luciferase-tagged MCMV at 1.0 × 10^5^ PFU intraperitoneal (i.p.) in a final volume of 100 μL PBS or remained mock-infected. Antiviral compounds were administered daily (d 0 to d 5) via oral gavage using feeding needles. A solution of 20% PHOSAL^®^ 50 PG (Lipoid GmbH, Ludwigshafen, Germany) in PBS was used as solvent and vehicle control. The control drug VGCV was administered in a dosage of 20 mg/kg/d as described previously [24], abemaciclib in dosages of 50 mg/kg/d and 100 mg/kg/d, and LDC4297 in a dosage of 20 mg/kg/d, where effectiveness against MCMV had previously been demonstrated in immunodeficient animals [31]. Mice were utilized for in vivo imaging at 4 d p.i., and sacrificed at 5 d p.i., after which spleen, liver and lung were dissected and stored at −80 °C. Experimental protocols were reviewed and approved by the Regierung von Unterfranken, Würzburg, Germany (permit 55.2-2532-2-416; 6 June 2017). 

Panel A of Figure 2 shows previously unpublished data of a mouse experiment detailed in Wild et al., 2020a [24]. The overall procedure was identical to the above, except for group size (5 mice), age of utilization (7 to 9 weeks) and time schedule (4 treatments, sacrifice at 4 d p.i.).

### 3.9. Organ Homogenization and In Vitro Luciferase Assay

For performing quantitative in vitro luciferase assays, frozen spleen, liver and lung tissues were prepared and homogenized in 1 mL Glo Lysis Buffer (Promega, Madison, WI, USA) using a Precellys 24 homogenizer (Bertin Technologies, Montigny le Bretonneux, France). Homogenates were centrifuged at 4 °C for 10 min at 14,000 rpm, and protein concentrations were determined using a Pierce™ BCA Protein Assay Kit (Thermo Fisher Scientific, Waltham, MA, USA). Determination of luciferase signals was performed in triplicates using 1 mM D-luciferin and an Orion II microplate luminometer (Berthold Technologies, Bad Wildbad, Germany). Mean of luciferase signal across 6 vehicle-treated animals was set as 100% in each organ.

### 3.10. DNA Extraction and Quantitative PCR

Extraction of DNA from spleen and liver samples was performed with the DNeasy^®^ Blood & Tissue kit (Qiagen, Hilden, Germany) according to the manufacturer’s protocol. Quantitative PCR was performed using an ABI Prism 7700 sequence detector (Applied Biosystems, Foster City, CA, USA) and the following oligonucleotides, 5′-MCMV (TGCCATACTGCCAGCTGAGA) and 3′-MCMV (GGCTTCATGATCCACCCTGTT) and the probe 5′-Fam/3′-BHQ1 (CTGGCATCCAGGAAAGGCTTGGTG) for the viral gene immediate early 1 (IE1). Assay was performed in triplicate, and mean of concentration-adjusted genome copy numbers across 6 vehicle-treated animals was set as 100% in each organ.

## Figures and Tables

**Table 1:** EC_50_ values determined for three preselected pharmaceutical kinase inhibitors (PKIs) against representatives of α-, β- and γ-herpesviruses.

	α-Herpesvirus	β-Herpesvirus		γ-Herpesvirus
	MDV ^a^	HCMV ^b^	MCMV ^c^	EBV ^d^
ABE	7.7 ± 5.6 µM	8.2 ± 4.1 µM	8.8 ± 0.1 µM	7.7 ± 0.9 µM
MBV	none (>100 µM)	0.56 ± 0.60 µM	none (>30 µM)	weak (>30 µM)
LDC4297	0.02 ± 0.01 µM ^e^	0.02 ± 0.00 µM ^e^	0.07 ± 0.02 µM ^e^	0.07 ± 0.06 µM

^a^ Plaque reduction assay, see Section 3.4 (antiviral effects additionally confirmed using qPCR; data not shown). ^b^ HCMV GFP-based replication assay, see Section 3.1 and Section 3.5. ^c^ MCMV GFP-based replication assay, see Section 3.1 and Section 3.7. ^d^ EBV GFP- based replication assay in Akata-BX-1 cells, see Section 3.3. ^e^ Sonntag et al., 2019 [31]. ABE, abemaciclib; MBV, maribavir.

**Table 2:** Combinatorial anti-CMV drug assessment using the Bliss independence checkerboard assay.

			95% Confidence Interval Synergy Volume [µM^2^%] ^b^		
Drug Combination	Cell Type/Virus	Replicates ^a^	Positive	Negative	Drug Interaction Type
MBV + GCV	HFF/HCMV AD169	2	**0.0 ± 0.0**	**−397.9 ± 39.2**	strongly antagonistic
ABE + GCV	HFF/HCMV AD169	2	**5.0 ± 7.0**	**−76.4 ± 56.1**	additive
ABE + LDC4297	HFF/HCMV AD169	2	**389.8 ± 117.1**	**− 4.5 ± 6.3**	strongly synergistic
ABE + MBV	HFF/HCMV AD169	2	**0.1 ± 0.1**	**− 67.7 ± 83.9**	additive
TF27 + GCV	HFF/HCMV AD169	2	**20.4 ± 3.6**	**− 19.6 ± 26.7**	additive
TF27 + LDC4297	HFF/HCMV AD169	1	**10.0**	**−6.7**	additive
TF27 + LMV	HFF/HCMV AD169	1	**61.1**	**−22.3**	additive
MBV + LDC4297	HFF/HCMV AD169	2	**138.6 ± 0.8**	**−1.5 ± 2.2**	strongly synergistic

^a^ Individual experimental replicates were performed with measurements in triplicate. ^b^ The synergy volume [µM^2^%] values defining the drug interaction type are in bold print. The types of drug interaction were set as follows: values below −100, strongly antagonistic; −100 to +50, additive; +50 to +100, moderately synergistic; above +100 strongly synergistic [57,58]. GCV, ganciclovir; LMV, letermovir.

**Table 3:** Combinatorial anti-CMV drug assessment using the Loewe additivity fixed-dose assay.

Drug Combination (EC_50_ Ratio)			EC_50_ [µM]		CI Values Extrapolated at % Virus Inhibition					
	Cell Type/Virus	Replicates ^a^	Drug A	Drug B	50	75	90	95	CI_wt_^b^	Type of Drug Interaction ^c^
MBV + GCV (1:1)	HFF/HCMV AD169	3	0.6 ± 0.9	1.5 ± 0.7	1.83 ± 1.46	1.81 ± 1.01	2.44 ± 0.45	4.00 ± 2.46	2.88 ± 0.83	antagonistic
ABE + GCV (1:1)	HFF/HCMV AD169	3	5.1 ± 2.8	1.8 ± 2.4	0.83 ± 0.38	0.64 ± 0.22	0.55 ± 0.09	0.55 ± 0.16	0.59 ± 0.08	synergistic
	MEF/MCMV	2	11.4 ± 3.5	2.5 ± 0.7	0.64 ± 0.01	0.71 ± 0.08	0.83 ± 0.14	0.94 ± 0.20	0.83 ± 0.14	moderately synergistic
ABE + LDC4297 (100:1)	HFF/HCMV AD169	2	6.2 ± 3.9	0.014 ± 0.018	3.40 ± 2.81	1.2 ± 0.24	0.70 ± 0.28	0.58 ± 0.35	1.03 ± 0.11	additive
GCV + LDC4297 (100:1)	HFF/HCMV AD169	3	0.9 ± 0.1	0.005 ± 0.003	1.25 ± 0.64	0.79 ± 0.18	0.59 ± 0.25	0.52 ± 0.31	0.66 ± 0.19	moderately synergistic
	MEF/MCMV	2	1.9 ± 1.3	0.086 ± 0.076	1.43 ± 0.78	0.98 ± 0.60	0.69 ± 0.43	0.54 ± 0.33	0.76 ± 0.46	moderately synergistic
TF27 + GCV (1:100)	HFF/HCMV AD169	2	0.036 ± 0.005	0.9 ± 0.6	2.49 ± 2.15	1.37 ± 0.53	1.16 ± 0.02	1.25 ± 0.13	1.37 ± 0.28	moderately antagonistic
MBV + LDC4297 (50:1)	HFF/HCMV AD169	2	0.5 ± 0.2	0.007 ± 0.0056	0.22 ± 0.06	0.30 ± 0.18	0.47 ± 0.35	0.64 ± 0.52	0.48 ± 0.35	synergistic
MBV + LDC4297 (100:1)	HFF/HCMV AD169	2	0.5 ± 0.7	0.007 ± 0.0012	0.26 ± 0.10	0.28 ± 0.02	0.40 ± 0.03	0.70 ± 0.31	0.48 ± 0.15	synergistic
	MEF/MCMV-UL97	1	1.3	0.004	0.57	0.46	0.38	0.33	0.40	synergistic
	ARPE-19/HCMV TB40	1	1.7	0.9	0.30	0.10	0.03	0.02	0.07	synergistic

^a^ Individual experimental replicates were performed with measurements in triplicate. ^b^ The CI_wt_ values defining the drug interaction type are in bold print and were calculated as (1 × CI_50_ + 2 × CI_75_ + 3 × CI_90_ + 4 × CI_95_)/10. ^c^ The types of drug interaction were set as follows: values <0.1 to 0.3, strongly synergistic; 0.3 to 0.7, synergistic; 0.7 to 0.85, moderately synergistic; 0.85 to 0.9, slightly synergistic; 0.90 to 1.10, (nearly) additive; 1.10 to 1.20, slightly antagonistic; 1.20 to 1.45, moderately antagonistic; 1.45 to 3.3, antagonistic; 3.3 to >10, strongly antagonistic [60].

## Data Availability

Data is contained within the article or Supplementary Material. Additional data is available on request from the corresponding author.

## Supplementary Materials

The following are available online at https://www.mdpi.com/1422-0067/22/2/575/s1.

## Abbreviations

ABE	abemaciclib
AIDS	acquired immunodeficiency syndrome
CDK	cyclin-dependent kinase
CDV	cidofovir
CI	combination index
CI_wt_	weighted combination index
CPE	cytopathic effect
d	day(s)
DAA	direct-acting antiviral(s)
DMEM	Dulbecco’s modified Eagle’s medium
DMSO	dimethyl sulfoxide
DNA	deoxyribonucleic acid
EBV	Epstein-Barr virus
EYFP	enhanced yellow fluorescent protein
FBS	fetal bovine serum
FOS	foscarnet
GCV	ganciclovir
GFP	green fluorescent protein
HCMV	human cytomegalovirus
HDA	host-directed antiviral(s)
HFF	human foreskin fibroblast
HIV	human immunodeficiency virus
LMV	letermovir
MBV	maribavir
MCMV	murine cytomegalovirus
MDV	Marek’s disease virus
MEF	murine embryonic fibroblast
MEM	Eagle’s Minimal Essential medium
MOI	multiplicity of infection
p.i.	post-infection
PBS	phosphate buffered saline
PFU	plaque-forming unit(s)
PKI	pharmaceutical kinase inhibitor
PRA	plaque reduction assay
qPCR	quantitative polymerase chain reaction
rpm	rotations per minute
SD	standard deviation
VGCV	valganciclovir

## References

[1] Cannon M.J., Schmid D.S., Hyde T.B. (2010). Review of cytomegalovirus seroprevalence and demographic characteristics associated with infection. Rev. Med. Virol..

[2] Mocarski E.S., Shenk T., Griffiths P.D., Pass R.F. (2013). Cytomegaloviruses. Fields Virology.

[3] Griffiths P., Baraniak I., Reeves M. (2015). The pathogenesis of human cytomegalovirus. J. Pathol..

[4] Sever J.L., Rakusan T.A., Ellaurie M., Frenkel N., Wyatt L.S., Campos J.M., O’Donnell R.M., Price M.V. (1995). Coinfection with herpesviruses in young children of HIV-infected women. Pediatr. AIDS HIV Infect..

[5] Meesing A., Razonable R.R. (2018). Pharmacologic and immunologic management of cytomegalovirus infection after solid organ and hematopoietic stem cell transplantation. Expert Rev. Clin. Pharmacol..

[6] Singh P., Neumann D.M. (2020). Persistent HCMV infection of a glioblastoma cell line contributes to the development of resistance to temozolomide. Virus Res..

[7] Revello M.G., Gerna G. (2002). Diagnosis and management of human cytomegalovirus infection in the mother, fetus, and newborn infant. Clin. Microbiol. Rev..

[8] Marschall M., Stamminger T. (2009). Molecular targets for antiviral therapy of cytomegalovirus infections. Future Microbiol..

[9] Lurain N.S., Chou S. (2010). Antiviral drug resistance of human cytomegalovirus. Clin. Microbiol. Rev..

[10] Marty F.M., Ljungman P., Chemaly R.F., Maertens J., Dadwal S.S., Duarte R.F., Haider S., Ullmann A.J., Katayama Y., Brown J. (2017). Letermovir Prophylaxis for Cytomegalovirus in Hematopoietic-Cell Transplantation. N. Engl. J. Med..

[11] Gerna G., Lilleri D., Baldanti F. (2019). An overview of letermovir: A cytomegalovirus prophylactic option. Expert Opin. Pharmacother..

[12] Marschall M., Stamminger T., Urban A., Wildum S., Ruebsamen-Schaeff H., Zimmermann H., Lischka P. (2012). In vitro evaluation of the activities of the novel anticytomegalovirus compound AIC246 (letermovir) against herpesviruses and other human pathogenic viruses. Antimicrob. Agents Chemother..

[13] Britt W.J., Prichard M.N. (2018). New therapies for human cytomegalovirus infections. Antivir. Res..

[14] Krishna B.A., Wills M.R., Sinclair J.H. (2019). Advances in the treatment of cytomegalovirus. Br. Med. Bull..

[15] Marty F.M., Ljungman P., Papanicolaou G.A., Winston D.J., Chemaly R.F., Strasfeld L., Young J.A., Rodriguez T., Maertens J., Schmitt M. (2011). Maribavir prophylaxis for prevention of cytomegalovirus disease in recipients of allogeneic stem-cell transplants: A phase 3, double-blind, placebo-controlled, randomised trial. Lancet Infect. Dis..

[16] Prichard M.N. (2009). Function of human cytomegalovirus UL97 kinase in viral infection and its inhibition by maribavir. Rev. Med. Virol..

[17] Prichard M.N., Gao N., Jairath S., Mulamba G., Krosky P., Coen D.M., Parker B.O., Pari G.S. (1999). A recombinant human cytomegalovirus with a large deletion in UL97 has a severe replication deficiency. J. Virol..

[18] Hamirally S., Kamil J.P., Ndassa-Colday Y.M., Lin A.J., Jahng W.J., Baek M.-C., Noton S., Silva L.A., Simpson-Holley M., Knipe D.M. (2009). Viral mimicry of Cdc2/cyclin-dependent kinase 1 mediates disruption of nuclear lamina during human cytomegalovirus nuclear egress. PLoS Pathog..

[19] Krosky P.M., Baek M.-C., Coen D.M. (2003). The human cytomegalovirus UL97 protein kinase, an antiviral drug target, is required at the stage of nuclear egress. J. Virol..

[20] Marschall M., Marzi A., aus dem Siepen P., Jochmann R., Kalmer M., Auerochs S., Lischka P., Leis M., Stamminger T. (2005). Cellular p32 recruits cytomegalovirus kinase pUL97 to redistribute the nuclear lamina. J. Biol. Chem..

[21] Milbradt J., Auerochs S., Sticht H., Marschall M. (2009). Cytomegaloviral proteins that associate with the nuclear lamina: Components of a postulated nuclear egress complex. J. Gen. Virol..

[22] Wolf D.G., Courcelle C.T., Prichard M.N., Mocarski E.S. (2001). Distinct and separate roles for herpesvirus-conserved UL97 kinase in cytomegalovirus DNA synthesis and encapsidation. Proc. Natl. Acad. Sci. USA.

[23] Hahn F., Niesar A., Wangen C., Wild M., Grau B., Herrmann L., Capci A., Adrait A., Couté Y., Tsogoeva S.B. (2020). Target verification of artesunate-related antiviral drugs: Assessing the role of mitochondrial and regulatory proteins by click chemistry and fluorescence labeling. Antivir. Res..

[24] Wild M., Bertzbach L.D., Tannig P., Wangen C., Müller R., Herrmann L., Fröhlich T., Tsogoeva S.B., Kaufer B.B., Marschall M. (2020). The trimeric artesunate derivative TF27 exerts strong anti-cytomegaloviral efficacy: Focus on prophylactic efficacy and oral treatment of immunocompetent mice. Antivir. Res..

[25] Wild M., Hahn F., Grau B., Herrmann L., Niesar A., Schütz M., Lorion M.M., Ackermann L., Tsogoeva S.B., Marschall M. (2020). The Artemisinin-Derived Autofluorescent Compound BG95 Exerts Strong Anticytomegaloviral Activity Based on a Mitochondrial Targeting Mechanism. Int. J. Mol. Sci..

[26] Oiknine-Djian E., Bar-On S., Laskov I., Lantsberg D., Haynes R.K., Panet A., Wolf D.G. (2019). Artemisone demonstrates synergistic antiviral activity in combination with approved and experimental drugs active against human cytomegalovirus. Antivir. Res..

[27] Cai H., Kapoor A., He R., Venkatadri R., Forman M., Posner G.H., Arav-Boger R. (2014). In vitro combination of anti-cytomegalovirus compounds acting through different targets: Role of the slope parameter and insights into mechanisms of Action. Antimicrob. Agents Chemother..

[28] Sepúlveda-Crespo D., Sánchez-Rodríguez J., Serramía M.J., Gómez R., De La Mata F.J., Jiménez J.L., Muñoz-Fernández M. (2015). Triple combination of carbosilane dendrimers, tenofovir and maraviroc as potential microbicide to prevent HIV-1 sexual transmission. Nanomedicine.

[29] Woollard S.M., Kanmogne G.D. (2015). Maraviroc: A review of its use in HIV infection and beyond. Drug Des. Dev. Ther..

[30] Hutterer C., Eickhoff J., Milbradt J., Korn K., Zeittrager I., Bahsi H., Wagner S., Zischinsky G., Wolf A., Degenhart C. (2015). A novel CDK7 inhibitor of the Pyrazolotriazine class exerts broad-spectrum antiviral activity at nanomolar concentrations. Antimicrob. Agents Chemother..

[31] Sonntag E., Hahn F., Bertzbach L.D., Seyler L., Wangen C., Müller R., Tannig P., Grau B., Baumann M., Zent E. (2019). In vivo proof-of-concept for two experimental antiviral drugs, both directed to cellular targets, using a murine cytomegalovirus model. Antivir. Res..

[32] Kim E.S. (2017). Abemaciclib: First Global Approval. Drugs.

[33] Tunkel A.R., Glaser C.A., Bloch K.C., Sejvar J.J., Marra C.M., Roos K.L., Hartman B.J., Kaplan S.L., Scheld W.M., Whitley R.J. (2008). The management of encephalitis: Clinical practice guidelines by the Infectious Diseases Society of America. Clin. Infect. Dis..

[34] Bacigalupo A., Bregante S., Tedone E., Isaza A., Van Lint M.T., Moro F., Trespi G., Occhini D., Gualandi F., Lamparelli T. (1996). Combined foscarnet -ganciclovir treatment for cytomegalovirus infections after allogeneic hemopoietic stem cell transplantation (Hsct). Bone Marrow Transpl..

[35] Mylonakis E., Kallas W.M., Fishman J.A. (2002). Combination antiviral therapy for ganciclovir-resistant cytomegalovirus infection in solid-organ transplant recipients. Clin. Infect. Dis..

[36] Sastry S.M., Epps C.H., Walton R.C., Rana S.N., Sanders R.J. (1996). Combined ganciclovir and foscarnet in pediatric cytomegalovirus retinitis. J. Natl. Med. Assoc..

[37] Jacquet C., Marschall M., Andouard D., El Hamel C., Chianea T., Tsogoeva S.B., Hantz S., Alain S. (2020). A highly potent trimeric derivative of artesunate shows promising treatment profiles in experimental models for congenital HCMV infection in vitro and ex vivo. Antivir. Res..

[38] Çapcı A., Lorion M.M., Mai C., Hahn F., Hodek J., Wangen C., Weber J., Marschall M., Ackermann L., Tsogoeva S.B. (2020). (Iso)Quinoline-Artemisinin Hybrids via Click Chemistry: Highly Potent Agents against Viruses. Chem. Eur. J..

[39] Marschall M., Niemann I., Kosulin K., Bootz A., Wagner S., Dobner T., Herz T., Kramer B., Leban J., Vitt D. (2013). Assessment of drug candidates for broad-spectrum antiviral therapy targeting cellular pyrimidine biosynthesis. Antivir. Res..

[40] Hahn F., Hutterer C., Henry C., Hamilton S.T., Strojan H., Kraut A., Schulte U., Schütz M., Kohrt S., Wangen C. (2018). Novel cytomegalovirus-inhibitory compounds of the class pyrrolopyridines show a complex pattern of target binding that suggests an unusual mechanism of antiviral activity. Antivir. Res..

[41] Hahn F., Wangen C., Häge S., Peter A.S., Dobler G., Hurst B., Julander J., Fuchs J., Ruzsics Z., Überla K. (2020). IMU-838, a Developmental DHODH Inhibitor in Phase II for Autoimmune Disease, Shows Anti-SARS-CoV-2 and Broad-Spectrum Antiviral Efficacy In Vitro. Viruses.

[42] Sonntag E., Hamilton S.T., Bahsi H., Wagner S., Jonjic S., Rawlinson W.D., Marschall M., Milbradt J. (2016). Cytomegalovirus pUL50 is the multi-interacting determinant of the core nuclear egress complex (NEC) that recruits cellular accessory NEC components. J. Gen. Virol..

[43] Feichtinger S., Stamminger T., Müller R., Graf L., Klebl B., Eickhoff J., Marschall M. (2011). Recruitment of cyclin-dependent kinase 9 to nuclear compartments during cytomegalovirus late replication: Importance of an interaction between viral pUL69 and cyclin T1. J. Gen. Virol..

[44] Hutterer C., Milbradt J., Hamilton S., Zaja M., Leban J., Henry C., Vitt D., Steingruber M., Sonntag E., Zeitträger I. (2017). Inhibitors of dual-specificity tyrosine phosphorylation-regulated kinases (DYRK) exert a strong anti-herpesviral activity. Antivir. Res..

[45] Hamilton S., Corina H., Egilmezer E., Steingruber M., Milbradt J., Korn K., Rawlinson W. (2018). Human cytomegalovirus utilises cellular dual-specificity tyrosine phosphorylation-regulated kinases during placental replication. Placenta.

[46] Hutterer C., Hamilton S., Steingruber M., Zeitträger I., Bahsi H., Thuma N., Naing Z., Örfi Z., Örfi L., Socher E. (2016). The chemical class of quinazoline compounds provides a core structure for the design of anticytomegaloviral kinase inhibitors. Antivir. Res..

[47] Steingruber M., Marschall M. (2020). The Cytomegalovirus Protein Kinase pUL97:Host Interactions, Regulatory Mechanisms and Antiviral Drug Targeting. Microorganisms.

[48] Biron K.K., Harvey R.J., Chamberlain S.C., Good S.S., Smith A.A., Davis M.G., Talarico C.L., Miller W.H., Ferris R., Dornsife R.E. (2002). Potent and selective inhibition of human cytomegalovirus replication by 1263W94, a benzimidazole L-riboside with a unique mode of action. Antimicrob. Agents Chemother..

[49] Koszalka G.W., Johnson N.W., Good S.S., Boyd L., Chamberlain S.C., Townsend L.B., Drach J.C., Biron K.K. (2002). Preclinical and toxicology studies of 1263W94, a potent and selective inhibitor of human cytomegalovirus replication. Antimicrob. Agents Chemother..

[50] Lalezari J.P., Aberg J.A., Wang L.H., Wire M.B., Miner R., Snowden W., Talarico C.L., Shaw S., Jacobson M.A., Drew W.L. (2002). Phase I dose escalation trial evaluating the pharmacokinetics, anti-human cytomegalovirus (HCMV) activity, and safety of 1263W94 in human immunodeficiency virus-infected men with asymptomatic HCMV shedding. Antimicrob. Agents Chemother..

[51] Ma J.D., Nafziger A.N., Villano S.A., Gaedigk A., Bertino J.S. (2006). Maribavir pharmacokinetics and the effects of multiple-dose maribavir on cytochrome P450 (CYP) 1A2, CYP 2C9, CYP 2C19, CYP 2D6, CYP 3A, N-acetyltransferase-2, and xanthine oxidase activities in healthy adults. Antimicrob. Agents Chemother..

[52] Marty F.M., Boeckh M. (2011). Maribavir and human cytomegalovirus-what happened in the clinical trials and why might the drug have failed?. Curr. Opin. Virol..

[53] Chou S., Waldemer R.H., Senters A.E., Michels K.S., Kemble G.W., Miner R.C., Drew W.L. (2002). Cytomegalovirus UL97 Phosphotransferase Mutations That Affect Susceptibility to Ganciclovir. J. Infect. Dis..

[54] Chou S. (2008). Cytomegalovirus UL97 mutations in the era of ganciclovir and maribavir. Rev. Med. Virol..

[55] Prichard M.N., Shipman C. (1990). A three-dimensional model to analyze drug-drug interactions. Antivir. Res..

[56] Chou T.-C., Talalay P. (1984). Quantitative analysis of dose-effect relationships: The combined effects of multiple drugs or enzyme inhibitors. Adv. Enzym. Regul..

[57] Evers D.L., Komazin G., Shin D., Hwang D.D., Townsend L.B., Drach J.C. (2002). Interactions among antiviral drugs acting late in the replication cycle of human cytomegalovirus. Antivir. Res..

[58] Wildum S., Zimmermann H., Lischka P. (2015). In vitro drug combination studies of Letermovir (AIC246, MK-8228) with approved anti-human cytomegalovirus (HCMV) and anti-HIV compounds in inhibition of HCMV and HIV replication. Antimicrob. Agents Chemother..

[59] Chou T.C., Martin N. (2005). CompuSyn for Drug Combinations: PC Soft-Ware and User’s Guide: A Computer Program for Quantitation of Synergism and Antagonism in Drug Combinations, and the Determination of IC50 and ED50 and LD50 Values.

[60] Chou T.C. (2006). Theoretical basis, experimental design, and computerized simulation of synergism and antagonism in drug combination studies. Pharmacol. Rev..

[61] Hutterer C., Niemann I., Milbradt J., Fröhlich T., Reiter C., Kadioglu O., Bahsi H., Zeitträger I., Wagner S., Einsiedel J. (2015). The broad-spectrum antiinfective drug artesunate interferes with the canonical nuclear factor kappa B (NF-κB) pathway by targeting RelA/p65. Antivir. Res..

[62] Reiter C., Fröhlich T., Gruber L., Hutterer C., Marschall M., Voigtländer C., Friedrich O., Kappes B., Efferth T., Tsogoeva S.B. (2015). Highly potent artemisinin-derived dimers and trimers: Synthesis and evaluation of their antimalarial, antileukemia and antiviral activities. Bioorg. Med. Chem..

[63] Hahn F., Fröhlich T., Frank T., Bertzbach L.D., Kohrt S., Kaufer B.B., Stamminger T., Tsogoeva S.B., Marschall M. (2018). Artesunate-derived monomeric, dimeric and trimeric experimental drugs—Their unique mechanistic basis and pronounced antiherpesviral activity. Antivir. Res..

[64] Wagner M., Michel D., Schaarschmidt P., Vaida B., Jonjic S., Messerle M., Mertens T., Koszinowski U. (2000). Comparison between human cytomegalovirus pUL97 and murine cytomegalovirus (MCMV) pM97 expressed by MCMV and vaccinia virus: pM97 does not confer ganciclovir sensitivity. J. Virol..

[65] Chou S., Ercolani R.J., Derakhchan K. (2018). Antiviral activity of maribavir in combination with other drugs active against human cytomegalovirus. Antivir. Res..

[66] Molesworth S.J., Lake C.M., Borza C.M., Turk S.M., Hutt-Fletcher L.M. (2000). Epstein-Barr virus gH is essential for penetration of B cells but also plays a role in attachment of virus to epithelial cells. J. Virol..

[67] Marschall M., Freitag M., Weiler S., Sorg G., Stamminger T. (2000). Recombinant green fluorescent protein-expressing human cytomegalovirus as a tool for screening antiviral agents. Antimicrob. Agents Chemother..

[68] Wagenknecht N., Reuter N., Scherer M., Reichel A., Müller R., Stamminger T. (2015). Contribution of the Major ND10 Proteins PML, hDaxx and Sp100 to the Regulation of Human Cytomegalovirus Latency and Lytic Replication in the Monocytic Cell Line THP-1. Viruses.

[69] Klenovsek K., Weisel F., Schneider A., Appelt U., Jonjic S., Messerle M., Bradel-Tretheway B., Winkler T.H., Mach M. (2007). Protection from CMV infection in immunodeficient hosts by adoptive transfer of memory B cells. Blood.

[70] Schumacher D., Tischer B.K., Fuchs W., Osterrieder N. (2000). Reconstitution of Marek’s disease virus serotype 1 (MDV-1) from DNA cloned as a bacterial artificial chromosome and characterization of a glycoprotein B-negative MDV-1 mutant. J. Virol..

[71] Bertzbach L.D., van Haarlem D.A., Härtle S., Kaufer B.B., Jansen C.A. (2019). Marek’s Disease Virus Infection of Natural Killer Cells. Microorganisms.

[72] Conradie A.M., Bertzbach L.D., Bhandari N., Parcells M., Kaufer B.B. (2019). A Common Live-Attenuated Avian Herpesvirus Vaccine Expresses a Very Potent Oncogene. mSphere.

