# Supplementary material for: Combinatorial Drug Treatments Reveal Promising Anticytomegaloviral Profiles for Clinically Relevant Pharmaceutical Kinase Inhibitors (PKIs)
Source: Int J Mol Sci. 2021 Jan 8;22(2):575. doi: 10.3390/ijms22020575 (PMC7826512; doi:10.3390/ijms22020575)
Supplement: Supplementary file 1 [file ijms-22-00575-s001.pdf]

International Journal of
Molecular Sciences

Supplementary Materials

Article

Combinatorial drug treatments reveal promising anticytomegaloviral profiles for clinically relevant pharmaceutical kinase inhibitors (PKIs)

Markus Wild[1], Jintawee Kicuntod[1], Lisa Seyler[2], Christina Wangen[1], Luca D. Bertzbach[3], Andelé M. Conradie[3], Benedikt B. Kaufer[3], Sabrina Wagner[1], Detlef Michel[4], Jan Eickhoff[5], Svetlana B. Tsogoeva[6], Tobias Bäuerle[2], Friedrich Hahn[1], Manfred Marschall[1,*]

1 Institute for Clinical and Molecular Virology, Friedrich-Alexander University of Erlangen-Nürnberg (FAU), Schlossgarten 4, 91054 Erlangen, Germany; markus.wild@uk-erlangen.de, jintawee.kicuntod@extern.uk-erlangen.de, christina.wangen@uk-erlangen.de, sabrina.wagner@uk-erlangen.de, friedrich.hahn@uk-erlangen.de, manfred.marschall@fau.de
2 Institute of Radiology, University Medical Center Erlangen, FAU, Palmsanlage 5, 91054 Erlangen, Germany; lisa.seyler@uk-erlangen.de, tobias.baeuerle@uk-erlangen.de
3 Institute of Virology, Freie Universität Berlin, Robert-von-Ostertag-Straße 7-13, 14163 Berlin, Germany; luca.bertzbach@fu-berlin.de; andele.conradie@fu-berlin.de; benedikt.kaufer@fu-berlin.de
4 Institute for Virology, Ulm University Medical Center, Albert-Einstein-Allee 11, Ulm, Germany; detlef.michel@uniklinik-ulm.de
5 Lead Discovery Center GmbH, Otto-Hahn-Str. 15, 44227 Dortmund, Germany; eickhoff@lead-discovery.de
6 Institute of Organic Chemistry I, FAU, Nikolaus-Fiebiger-Straße 10, 91058 Erlangen Germany; svetlana.tsogoeva@fau.de
* Correspondence: manfred.marschall@fau.de; Tel.: ++49 9131 8526089

Received: date; Accepted: date; Published: date

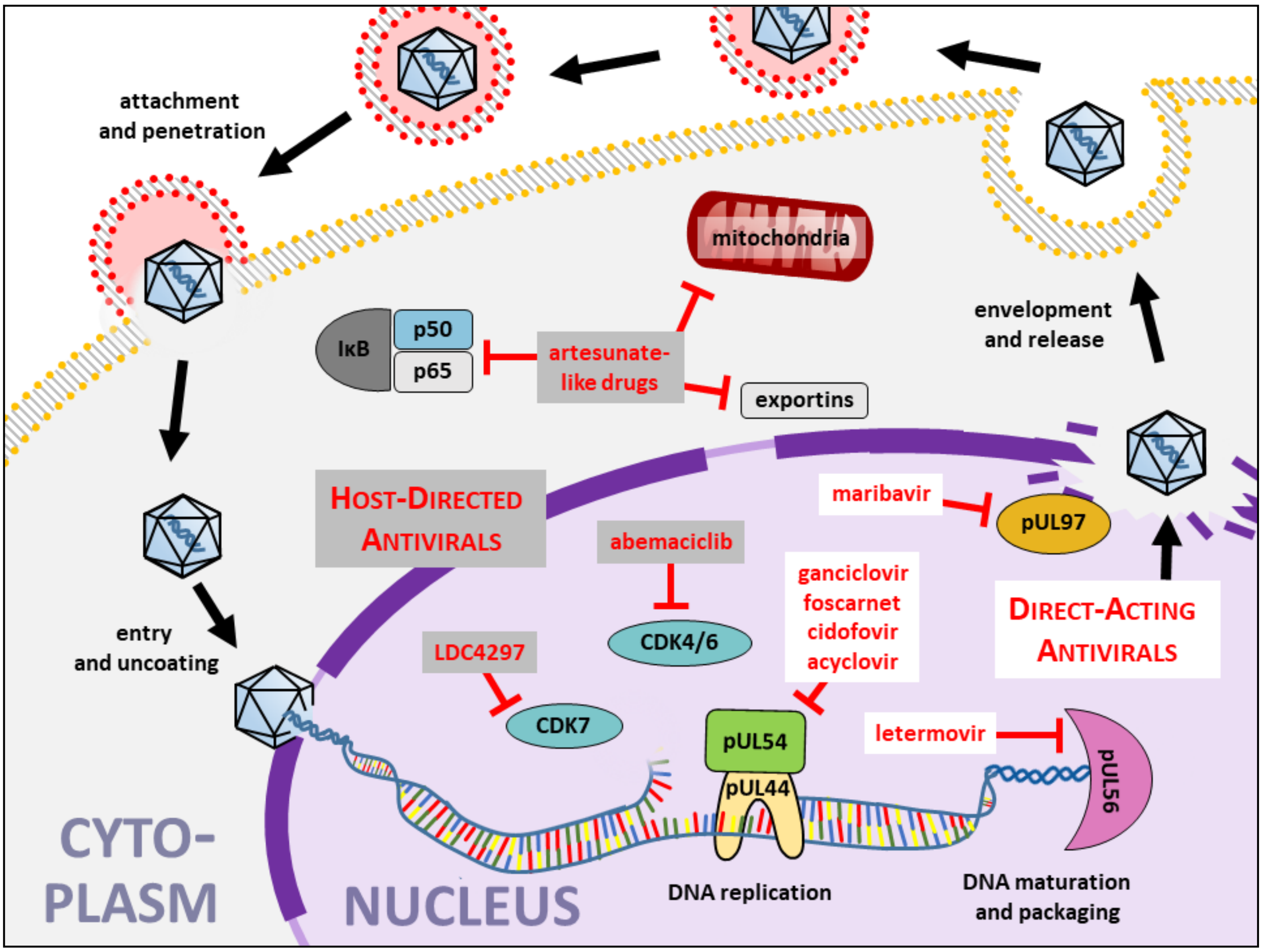

Figure S1. Scheme of the HCMV replication cycle illustrating the distinct modes of action of approved direct-acting antivirals (DAAs; i.e. ganciclovir, foscarnet, cidofovir, acyclovir, letermovir), developmental DAA (maribavir) and host-directed antivirals (HDAs; i.e. artesunate-like drugs, abemaciclib, LDC4297).
